# Impact of underground storm drain systems on larval ecology of *Culex* and *Aedes* species in urban environments of Southern California

**DOI:** 10.1038/s41598-021-92190-3

**Published:** 2021-06-16

**Authors:** Xiaoming Wang, Guofa Zhou, Daibin Zhong, Yiji Li, Stacia Octaviani, Andrew T. Shin, Timothy Morgan, Kiet Nguyen, Jessica Bastear, Melissa Doyle, Robert F. Cummings, Guiyun Yan

**Affiliations:** 1 Program in Public Health, University of California, Irvine, CA 92697-4050 USA; 2 Orange County Mosquito and Vector Control District, Garden Grove, CA 92843 USA; 3 San Gabriel Valley Mosquito and Vector Control District, West Covina, CA 91790 USA

**Keywords:** Entomology, Invasive species

## Abstract

An extensive network of storm water conveyance systems in urban areas, often referred to as the “underground storm drain system” (USDS), serves as significant production habitats for mosquitoes. Knowledge of whether USDS habitats are suitable for newly introduced dengue vectors *Aedes aegypti* and *Ae. albopictus* will help guide surveillance and control efforts. To determine whether the USDS functions as a suitable larval habitat for *Culex*, *Ae. aegypti* and *Ae. albopictus* in southern California, we examined mosquito habitat utilization and larval survivorship using laboratory microcosm studies. The data showed that USDS constituted 4.1% of sampled larval habitats for *Ae. aegypti* and *Ae. albopictus,* and 22.0% for *Cx. quinquefasciatus*. Furthermore, USDS water collected in the summer completely inhibited *Aedes* larval development, but yielded a 15.0% pupation rate for *Cx. quinquefasciatus.* Food supplementation in the microcosms suggests that nutrient deficiency, toxins and other factors in the USDS water led to low success or complete failure of larval development. These results suggest that USDS habitats are currently not major productive larval habitats for *Aedes* mosquitoe*s* in southern California. Our findings prompt inclusion of assessments of pupal productivity in USDS habitats and adult mosquito resting sites in the mosquito surveillance program.

## Introduction

Rapid urbanization in the past several decades have altered microclimatic conditions and natural ecology, which may subsequently affect the ecology of disease vectors and risk of mosquito-borne diseases in heavily populated landscapes^1–3^. For example, more mosquito larval habitats and higher abundance of the yellow fever mosquito *Aedes* *aegypti*, the most important vector of dengue, chikungunya, and Zika viruses, were found in urban areas than in suburban and rural areas in Côte d’Ivoire, Africa^4^. Similarly, not only were more larval habitats found for the Asian tiger mosquito, *Aedes albopictus*, in urban areas of southern China, *Aedes* larvae developed faster and the adult emergence rate was higher than in suburban and rural areas, partly due to higher ambient temperatures associated with heat islands in urban areas^5^.

*Aedes aegypti* and *Ae. albopictus* are considered as the most invasive mosquitoes in the world^6,7^. In the state of California, USA, *Ae. albopictus* was introduced in 2001 and considered established in 2011, while *Ae. aegypti* was discovered in 2013^8^. In southern California, common aquatic mosquito habitats include peridomestic sources (e.g., small artificial water-holding containers, unmaintained ornamental ponds and swimming pools)^9^, and an extensive network of storm water conveyance systems consisting of catch basins, manhole chambers, underground vaults, pipelines and tunnels, and is collectively referred to as the “underground storm drain system (USDS)”^10^.

The USDS is designed to rapidly direct water from heavy rainstorms to large channels to slow runoff and reduce erosion, improve water quality and avoid flooding of streets, homes, and businesses. However, non-stormwater runoff into the USDS from landscape irrigation of residential and commercial establishments, as well as highways and streets, in highly populated southern California is tremendous. The USDS has been documented as suitable, year-round larval habitats for *Culex quinquefasciatus* mosquitoes at times and places in the system where subsurface water drainage is slow and irregular, as often by design or damage^10,11^. In addition, the arid climate of southern California can desiccate small containers rapidly, so large numbers of stable aquatic habitats in the USDS may pose very significant challenges for the control of *Aedes*-transmitted viruses if these habitats are suitable to egg laying and larval development for invasive *Aedes*. However, because aquatic habitats in the USDS tend to have low dissolved oxygen, above normal electrical conductivity and salinity levels^10^, and perhaps residual pesticides from dry-weather runoff during summer months^12^, USDS water may inhibit larval development and oviposition choice by gravid female mosquitoes.

Larval habitats in the USDS are extremely difficult to access and apply pesticides for mosquito control. A better understanding of the larval ecology and pupal productivity of invasive *Aedes* mosquitoes in USDS aquatic habitats will facilitate the development of rational control strategies. Given the rapid spread of *Ae. aegypti* and *Ae. albopictus* in many regions of the world^13–15^, information on the larval ecology of invasive *Aedes* mosquitoes in USDS water will be useful in assessing the impact of environmental changes on the risk of arboviruses transmitted by these exotic *Aedes* mosquitoes in southern California. The objective of this study was to assess habitat utilization by *Ae. aegypti* and *Ae. albopictus* following their discovery in Orange County, California, in 2015 and to examine whether the USDS provides suitable habitats for *Ae. aegypti* and *Ae. albopictus* larvae through life table studies in controlled microcosms and extensive field surveys*.* We investigated: (1) habitat usage by invasive *Aedes* mosquitoes from 2016–2019, (2) suitability of the USDS habitat for invasive *Aedes* egg laying, hatching, and larval development, and (3) impact of *Cx. quinquefasciatus* larvae, the predominant mosquito species in urban southern California^16,17^, on *Aedes* oviposition preference and larval survival.

## Methods

### Ethics and vertebrate animals

The field surveys and collections were conducted on accessible public areas or private residential areas with property owners’ permission. The study did not involve human participants, or endangered or protected species. Laboratory mice were used as a blood source for mosquitoes. All experimental protocols were approved by the Institutional Animal Care and Use Committee (IACUC) of the University of California, Irvine (UCI) (IACUC protocol number: AUP-19-165). All methods were carried out in accordance with relevant IACUC guidelines and regulations.

### Study sites and mosquito larval habitat surveillance

The study was carried out in Orange County, California, USA. Orange County is a highly urbanized county with an estimated population density of approximately 1470 people/km^2^ according to U.S. Census Bureau, an average annual low/high temperature range of 13–25 °C, 65% relative humidity, and annual precipitation of about 350 mm according to U.S. Climate Data. Annual rainfall was 261 mm, 311 mm, 198 mm and 475 mm for 2016, 2017, 2018 and 2019, respectively. A major drought event occurred in December 2017 and February 2018 when the total rainfall in the 3-month period was 20.6% of the 30-year average. Both *Ae. aegypti* and *Ae. albopictus* were discovered in the county in 2015^8^. *Culex quinquefasciatus* is the most abundant mosquito in the county and breeds readily in a variety of residential, commercial and USDS water sources, and is the primary vector of West Nile virus in southern California^18^.

Larval mosquito surveillance in Orange County was conducted from 2016 to 2019 by the Orange County Mosquito and Vector Control District (OCMVCD) through its routine mosquito surveillance and treatment program, following the recommendations of the California Department of Public Health and the Mosquito and Vector Control Association of California^19^. Briefly, OCMVCD staff conducted routine inspection for aquatic habitats in randomly selected public areas, and performed door-to-door mosquito larval and adult sampling on residential or commercial premises upon the request of the residents or business owners while distributing public education materials for vector control and personal protection. Arial photography was used to examine the presence of abandoned swimming pools in residential areas. In addition to surface aquatic habitats, subsurface habitats (e.g., catch basins, underground drains, manhole chambers, and public utility vaults) were examined for larval abundance of all mosquito species. In 2019, OCMVCD completed 5,622 mosquito service requests, and conducted 11,813 inspection and treatments on routine sites using a variety of public health-approved adulticides and larvicides. A total of 38,099 underground drains and catch basins and 6925 km of flood channels were treated. In addition, a total of 17,783 km of gutters and 3562 neglected swimming pools were inspected and treated. The larval distribution data reported here were based on this extensive field sampling effort^20^.

Larval sampling used standard mosquito dippers or pipettes, and specialized modifications of these to sample hard to reach areas. Mosquito larvae from each source were collected, transferred into a uniquely-numbered vial with isopropyl alcohol (70%), and submitted to the laboratory for identification; if present, live pupae were collected and held in site-specific labelled rearing chambers (BioQuip Products, Inc., Rancho Dominguez, CA) until emergence. Third and fourth instar mosquito larvae (1–100, depending on sample size) and emerged adults were identified to species using a stereo microscope (40–50x) and morphological features described in taxonomic keys^21,22^. Results were uploaded to OCMVCD’s data management system, along with collection date, GPS location, and habitat type for each sample site. For this study, larval habitats were classified into six types: small container, underground system, ornamental water features, marsh, pools/spas, and creek (Table S1). The container classification included flowerpots/vases, saucers, tires, bowls, boxes, buckets, dishes, tree holes, etc. Underground storm drain system referred to larval habitats such as catch basins, manhole chambers, underground drains, and public utility vaults that were below the ground. Water feature included flood control channels, ponds, fountains, birdbaths, street gutters and small reservoirs, etc. Marsh included both fresh and salt water marshes.

### Mosquito strains and water source for laboratory studies

We examined the effect of USDS water on oviposition substrate preference and larval development in microcosms in an insectary with climate control (27 ± 1 °C, 70 ± 10% relative humidity, and 12 h light/12 h dark photoperiod) at UCI. To minimize potential bias on behavior and ecology from mosquito colonization, this study did not use previously established laboratory mosquito colonies. Instead, we used *Ae. aegypti* and *Ae. albopictus* adults reared from field-collected eggs using ovicups in residential areas of Orange and Los Angeles Counties, California, respectively. *Culex quinquefasciatus* were also reared from eggs of field-collected, blood-engorged adult mosquitoes using gravid traps in Orange County^23^.

All experiments reported here used two types of habitat water: (1) USDS water collected from seven manhole chambers or catch basins (33°47′01.9"N, 117°53′19.0"W, Orange City, manhole; 33°52′25.0"N, 117°57′02.6"W, Fullerton City, manhole; 33°44′44.4"N, 118°06′24.2"W, Seal Beach City, manhole; 33°55′38.9"N, 117°56′51.4"W, La Habra City, manhole; 33°52′48.9"N, 117°55′21.4"W, Fullerton City, catch basin; 33°54′35.2"N, 117°56′02.5"W, Fullerton City, catch basin; 33°52′25.0"N, 117°57′02.6"W, Fullerton City, catch basin); and 2) flowerpot water from vases of three cemeteries in Orange County (33°50′29.0"N, 117°53′57.9"W; 33°46′21.5"N, 117°50′35.8"W; 33°46′12.3"N, 117°50′21.4"W). Water (including sediments) from each breeding source was collected with mosquito dippers and mixed together by habitat type into 18.9 L (five-gallon) Nalgene™ containers. The containers were transported to the laboratory in shaded ice containers, and stored overnight in a refrigerator at 4 °C. The experiments described below were conducted on the field-collected water for the two habitat types. We selected flowerpot water as the comparison substrate because flowerpot containers showed the highest larval positivity rate in the study area.

### Oviposition preference test

To examine whether USDS water attracts or repels egg laying by *Ae. aegypti* and *Ae. albopictus* mosquitoes, a two-choice oviposition preference test was conducted. Briefly, this experiment used two ovicups placed within a mosquito cage (1 × 0.5 × 0.5 m^3^), one ovicup with 200 ml USDS water and another with 200 ml flowerpot water. Adult mosquitoes were bloodfed on mice; fully engorged females 3-days post-bloodfeeding were used for oviposition preference tests. Ten gravid *Ae. aegypti* females were released into a cage and allowed to lay eggs for three days, and the number of eggs in each ovicup were counted. Five replicates were used. The same experiment was conducted for *Ae. albopictus*.

To evaluate whether the presence of *Cx. quinquefasciatus* larvae has any impact on the egg laying behavior of invasive *Aedes* mosquitoes, the two-choice oviposition preference test described above was used. One ovicup contained 200 ml USDS water and ten first-instar *Cx. quinquefasciatus* larvae, while the second ovicup contained 200 ml USDS water only. Ten gravid *Ae. aegypti* or *Ae. albopictus* females were released into a cage and allowed to lay eggs for three days. Five replicates were used. We also conducted this experiment using flowerpot water with the same design and same number of replicates to determine whether the impact of *Cx. quinquefasciatus* larvae on *Aedes* mosquito egg laying behavior was similar across different water substrate types.

### Egg hatching

To investigate the effects of different habitat water sources on egg hatching, 50 *Ae. aegypti* or *Ae. albopictus* eggs on separate filter papers were introduced into ovicups with 200 ml USDS water or flowerpot water. Deoxygenized distilled water that we routinely use in laboratory mosquito colony maintenance was used as a positive control. The experiment was conducted in an insectary with climate control (27 ± 1 °C). The number of larvae hatched were counted daily for six days continuously. Five replicates were used.

### Larval survivorship

A life table study was conducted on *Ae. aegypti* and *Ae. albopictus* larvae to determine the effect of USDS water and flowerpot water on larval development and survivorship. Twenty-five newly hatched *Ae. aegypti* or *Ae. albopictus* larvae were introduced into a microcosm that contained 200 ml USDS or field flowerpot water. The number of dead and surviving larvae was recorded daily until they pupated. Pupae were counted, and removed to different paper cups for emergence to adults. Four replicates were used for each type of habitat water per species. We included *Cx. quinquefasciatus* in the larval life table study for method validation purposes because the larvae of this species were known to successfully develop into pupae and adults in USDS water in southern California^10^.

Larval survivorship experiments were conducted in two different seasons. The first was in the summer (August–September) 2019 when the density of invasive *Aedes* species peaked^19^, and also insecticide runoff from mosquito and residential/agricultural pest control applications were at the highest levels in southern California^24^. The second was in the winter (December) 2019 when there was little insecticide treatment for mosquito and pest control. This design enabled us to examine seasonality in larval survivorship and the impact of environmental insecticide runoff in USDS water. To determine whether USDS water’s nutritional deficiency plays a major role in limiting *Aedes* larval development, we repeated the larval survival experiment by adding 0.1 g Tetramin Tropical Flakes, the standard larval mosquito diet in insectaries, to the microcosms every 2 days. The number of dead and surviving larvae, pupae, and emergent adults was recorded daily.

### Data analysis

All aquatic habitats that were positive or negative for the larvae of *Ae. aegypti*, *Ae. albopictus* and *Cx. quinquefasciatus* (the predominant species), were mapped using ArcGIS 10.7.1. The proportion of aquatic habitats positive for *Ae. aegypti* and *Cx. quinquefasciatus* was calculated for each habitat type from 2016 to 2019. To examine variation in *Aedes* and *Culex* larval positivity rate among different groups of larval habitats within the USDS, larval positivity rates for *Ae. aegypti* and *Cx. quinquefasciatus* were calculated for underground water retention vaults, underground catch basins/manholes, and underground pipelines/tunnels. The Chi-square test was used to examine the statistical significance. *Culex quinquefasciatus* was analyzed because it was the most common species, whereas *Ae. albopictus* was not included in the analysis due to insufficient number of *Ae. albopictus* positive habitats. To determine whether USDS water attracted or repelled oviposition of invasive *Aedes* mosquitoes, a pairwise *t* test was used to compare egg number in USDS water ovicups to flowerpot water ovicups for each *Aedes* species. Similarly, a pairwise *t*-test was used to test the effect of *Cx. quinquefasciatus* larvae on *Aedes* mosquito oviposition choice.

To examine the effect of water sources on egg hatching, the *t-test* was used to analyze the egg hatching rate. The analysis of larval life table study data focused on pupation rates and larval-to-pupal development times. The pupation rate was calculated as the proportion of first-instar larvae that molted into pupae. The effect of water sources and larval food supplementation on pupation rate was analyzed using non-parametric Wilcoxon test. The *t-test* was used to analyze the duration of larval-to-pupal development. Kaplan–Meier survival analysis was used to determine the effects of food supplementation and water source on larval development for each species, and the log-rank test was conducted to determine their statistical significance. All statistical analyses were performed using JMP software (JMP 14.2, SAS Institute Inc.).

## Results

### Ecological characterization of mosquito larval habitats

A total of 6,072 records with GPS coordinates, presence or absence of mosquito larvae and habitat type classification, were analyzed from 2016 to 2019 (Table S1). Within each sampling year, a majority (88.2% on average) of the larval habitats were collected once, 7.8% twice, and 4.1% more than 2 times. A total of 21 mosquito species across four genera were identified, including nine *Aedes* species, two *Anopheles* species, eight *Culex* species, and two *Culiseta* species (Table S2). *Culex quinquefasciatus*, *Ae. aegypti*, *Cx. tarsalis*, and *Culiseta incidens* were the dominant larval species, with occurrence constituting 65.5%, 18.3%, 17.1% and 12.8%, respectively, of the total 6072 larval collections. Of the six aquatic habitat types, containers, water features and USDS were the most common larval habitats for mosquitoes, comprising 31.4%, 24.8% and 17.4% respectively, of total aquatic habitats positive for any mosquito species.

**Figure 1:**
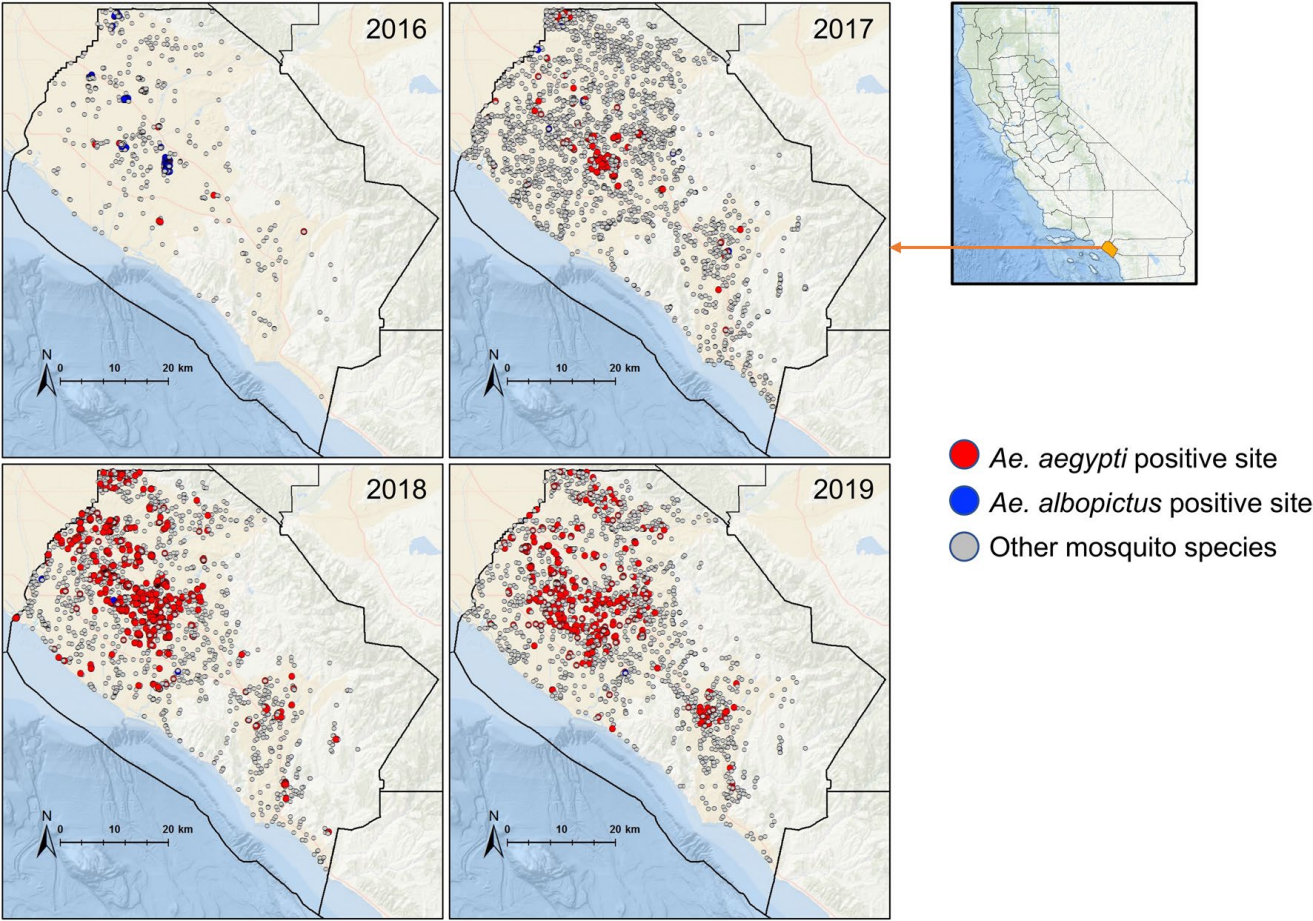
Maps of mosquito larval sampling sites in Orange County, California, from 2016 to 2019, showing the distribution of *Aedes aegypti* and *Ae. albopictus* positive larval habitats. The maps were generated using ArcGIS 10.7.1 (ESRI, USA).

*Aedes aegypti* and *Ae. albopictus,* the two focal species of the present study, exhibited differences in abundance and spatial distribution (Fig. 1). Over the four-year study period, *Ae. aegypti* was found in 18.3% (1,111/6,072) and *Ae. albopictus* was detected in 0.4% (25/6,072) of the collections (*χ*^*2*^ = 1,145.3; *d.f.* = 1, *P* < 0.0001), clearly demonstrating *Ae. aegypti* was more abundant than *Ae. albopictus* in the study area. The distribution map of these two invasive *Aedes* species showed that *Ae. aegypti* was spreading rapidly over time (Fig. 1). The biggest expansion period occurred between 2017 and 2018. The total number of habitats positive for *Ae. aegypti* each year was 48 (9.2%; total number of habitats n = 520), 110 (5.2%; n = 2099), 570 (33.1%; n = 1718) and 383 (22.1%; n = 1735) between 2016 and 2019; and 13 (2.5%), 6 (0.3%), 5 (0.3%), and 1 (0.1%) for *Ae. albopictus*. The overall habitat positivity rate of *Ae. aegypti* among all aquatic habitats exhibited an increasing trend from 9.2% in 2016 to 22.1% in 2019.

**Figure 2:**
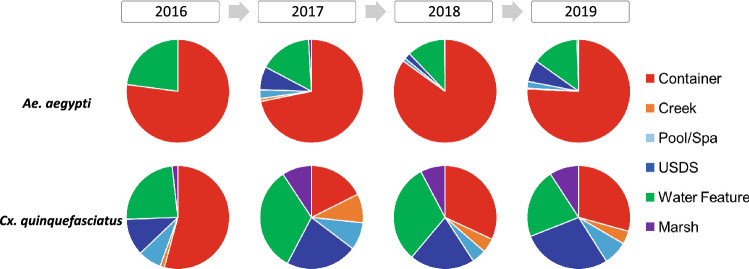
Dynamics of habitat utilization of *Aedes aegypti* and *Culex quinquefasciatus* mosquitoes from 2016 to 2019 in Orange County, California.

**Figure 3:**
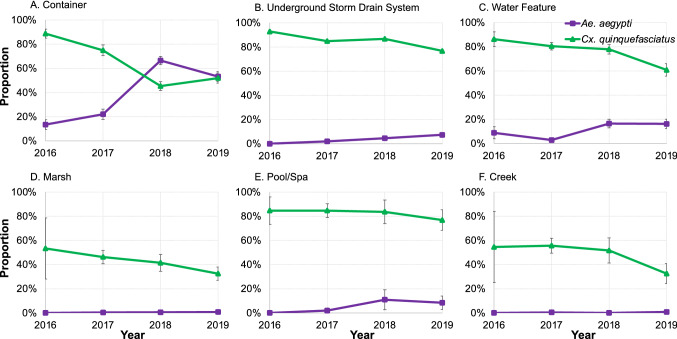
Positivity rates of *Aedes aegypti* and *Culex quinquefasciatus* larvae in six types of aquatic habitats from 2016 to 2019 in Orange County, California. (**A**) Container; (**B**) Underground storm drain system; (**C**) Water feature; (**D**) Marsh; (**E**) Pool/Spa; and (**F**) Creek. Positivity rate was calculated as the proportion of larval habitats positive for *Ae. aegypti* or *Cx. quinquefasciatus* larvae among all habitats examined.

In terms of habitat utilization, containers were the most common habitats for *Ae. aegypti* (Fig. 2). Among all *Ae. aegypti* positive habitats (n = 1111), 80.1% were classified as containers, 13.8% water features, and only 4.1% USDS over the four-year study (Fig. 3). Although USDS sources constituted a small proportion of larval habitats for *Ae. aegypti*, habitat positivity rate in the USDS exhibited an increasing trend (Fig. 3). Other habitat types showing an increasing trend of *Ae. aegypti* larval positivity included containers, water features, and pools/spas, probably due to increasing abundance of *Ae. aegypti* in the study area (Fig. 3). Among *Ae. albopictus* positive habitats (n = 25), 88.0% were classified as containers, 8.0% water features and 4.0% marsh. In comparison, containers and USDS sources were consistently the main habitats of larval *Cx. quinquefasciatus*, constituting 28.3% and 22.0% of mosquito-positive habitats during the four-year study (Fig. 2). The habitat positivity rate of *Cx. quinquefasciatus* exhibited a decreasing trend in all six habitat types (Fig. 3).

Because USDS habitats in our study represented several groups of underground aquatic habitats (underground water retention vaults, underground catch basins/manholes, and underground pipelines/tunnels), we examined whether these groups of aquatic habitats differed in mosquito larval positivity rate. A particularly interesting question is whether some USDS structures (e.g., catch basins) that are prone to collect debris and concentrate organic materials, which can serve as potential larval food sources, had a higher positivity rate for *Aedes* mosquito larvae. We found significant variations in *Ae. aegypti* positivity rate among the USDS habitat groups (*χ*^2^ = 15,6, *d.f.* = 2, *P* < 0.001), with the lowest positivity rate in the underground catch basins and manholes (0%), and the highest in the underground pipelines/tunnels (5.8%) (Table S3). In contrast, underground catch basins and manholes showed the highest positivity rate for *Cx. quinquefasciatus* (96.8%), whereas underground water retention vaults exhibited the lowest positivity rate (52.6%) (Table S3; *χ*^2^ = 63.7, *d.f.* = 2, *P* < 0.0001). Overall, these results demonstrated a low *Ae. aegypti* larval positivity rate but a high *Cx. quinquefasciatus* positivity rate in USDS aquatic habitats.

### Laboratory oviposition preference

**Figure 4:**
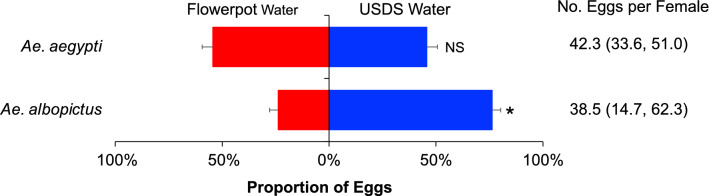
Oviposition substrate preference of *Aedes aegypti* and *Ae. albopictus* mosquitoes in a two-choice oviposition preference test with waters from underground storm drain systems and flowerpots.

The total numbers of eggs laid by *Ae. aegypti* and *Ae. albopictus* were similar (42.3 vs. 38.5 per female, *t* = 0.42, *d.f.* = 1, *P* > 0.05; Fig. 4). *Aedes albopictus* mosquitoes preferred USDS water for egg laying over the flowerpot water (*t* = 2.88, *d.f.* = 1, *P* < 0.05), but *Ae. aegypti* exhibited no preference for any aquatic substrate (Fig. 4). Water from USDS did not attract or repel *Ae. aegypti* in their egg laying choice.

In southern California, *Culex* larvae were present in the majority of containers and USDS habitats, and reached up to 80% habitat positivity, as shown in Fig. 3. It is thus interesting to examine whether the presence of *Culex* larvae attracted or repelled egg laying by the invasive *Aedes* species. We found that the presence of *Cx. quinquefasciatus* larvae did not attract or repel egg laying by *Ae. aegypti* in USDS or flowerpot water, and did not have a significant impact on the number of eggs laid in either type of oviposition substrate (Fig. S1A). A similar result was found for *Ae. albopictus* (Fig. S1B).

### Egg hatching

**Figure 5:**
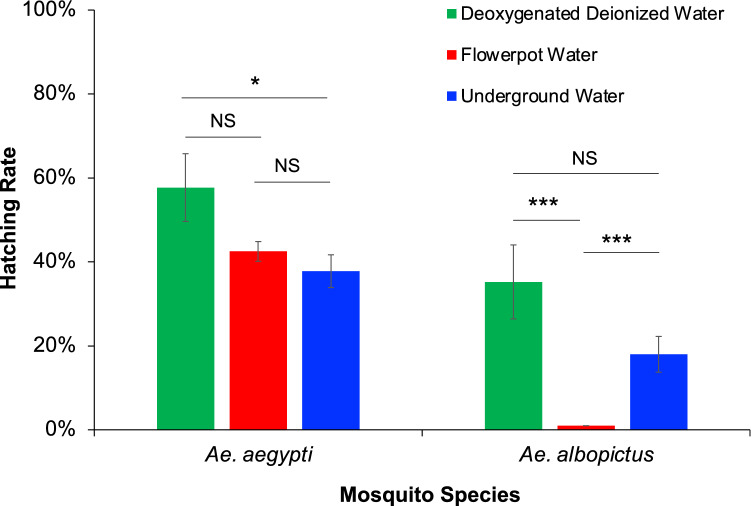
Egg hatching rate of *Aedes aegypti* and *Ae. albopictus* in waters collected in summer 2019, from underground storm drain systems and flowerpots in Orange County, California. The error bar indicates standard error.

The egg hatching rate of *Ae. aegypti* in USDS water was 37.8%, similar to the 42.5% hatching rate observed in the flowerpot water (Fig. 5). In contrast, the *Ae. albopictus* egg hatching rate was 1.0% in flowerpot water, significantly lower than the 18.0% hatching rate observed in USDS water. The low hatching rate of *Ae. albopictus* eggs in USDS water cannot be attributed to poor egg quality or other unsuitable environmental conditions because the same batch of *Ae. albopictus* eggs exhibited a 35.3% hatching rate in the deionized water under the same environmental conditions (Fig. 5).

### Larval survivorship

**Figure 6:**
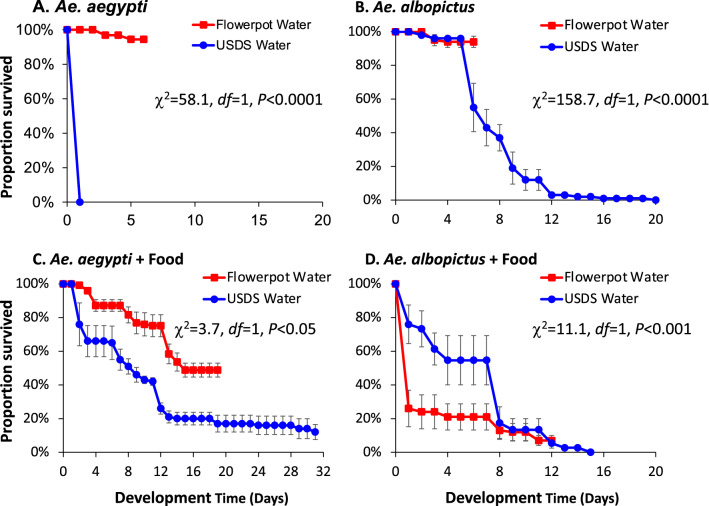
Survival curve of *Aedes aegypti* and *Ae. albopictus* larvae in microcosms with waters collected in summer, 2019, from underground storm drain systems and flowerpots in Orange County, California. (**A**) *Ae. aegypti* with waters from underground storm drain systems and flowerpots without food supplementation; (**B**) *Ae. albopictus* with waters from underground storm drain systems and flowerpots without food supplementation; (**C**) *Ae. aegypti* with waters from underground storm drain systems and flowerpots with food supplementation; (**D**) *Ae. albopictus* with waters from underground storm drain systems and flowerpots with food supplementation. Chi-squared and *P*-value of Kaplan–Meier survival analysis log-rank test is shown. The error bar indicates standard error.

To determine whether USDS water was suitable for larval development of the invasive *Aedes* mosquitoes, larval-to-pupal survivorship was examined in microcosms under controlled insectary conditions. The first experiment was conducted in summer 2019 using field-collected USDS water and flowerpot water. Both *Ae. aegypti* and *Ae. albopictus* larvae exhibited high pupation rate (> 94%) and fast larva-to-pupa development (7–8 days) in the control (flowerpot water, Table 1), suggesting the flowerpot water fully met the nutritional needs of *Aedes* larvae and supported their development. On the other hand, *Ae. aegypti* and *Ae. albopictus* larvae exhibited a zero pupation rate in the USDS water (Table 1). *Aedes aegypti* larvae died within two days after they were placed in the microcosms with USDS water (Fig. 6A). *Aedes albopictus* larvae developed very slowly in the USDS water, and none successfully developed into pupae (Fig. 6B). The pupation rate pattern was very similar between *Ae. aegypti* and *Ae. albopictus* (Table 1), suggesting that summer USDS water was not suitable for larval development for the two invasive *Aedes* species.

**Table 1:** Pupation rate and larval-to-pupal development duration of *Aedes aegypti, Ae. albopictus* and *Culex quinquefasciatus* larvae in microcosms with waters collected from flowerpots and underground storm drain systems, summer 2019.

Mosquito species	Habitat water type	No larval food supplementation				With larval food supplementation			
		Pupation rate (%)	*P*	Pupation time (days)	*P*	Pupation rate (%)	*P*	Pupation time (days)	*P*
*Ae. aegypti*	Flowerpot Water	94.4 (91.3, 97.5)	< 0.0001	7.5 (7.1, 7.9)	–	48.8 (39.7, 57.9)	< 0.01	11.6 (11.0, 12.1)	> 0.05
	Underground Water	0		–		12.0 (1.9, 22.1)		13.2 (11.3, 15.1)	
*Ae. albopictus*	Flowerpot Water	94.0 (86.5, 100)	0.0001	8.1 (7.9, 8.2)	–	7.0 (0.3, 13.7)	> 0.05	13.7 (13.4, 14.1)	–
	Underground Water	0		–		0		–	
*Cx. quinquefasciatus*	Flowerpot Water	82.0 (70.2, 93.8)	0.001	8.9 (8.7, 9.2)	0.0001	71.0 (62.3, 79.7)	> 0.05	8.2 (8.0, 8.5)	0.001
	Underground Water	15.0 (1.9, 28.1)		13.7 (13.2, 14.3)		81.3 (62.7, 99.9)		8.9 (8.7, 9.3)	

Note. Two treatments were conducted: the first used water from natural aquatic habitats from the field, and the second treatment was supplemented with larval food. Numbers in the bracket indicate 95% confidence interval. “-” indicates the parameter cannot be calculated.

We conducted additional experiments to determine whether complete inhibition of larval development in invasive *Aedes* mosquitoes in the summer USDS water was due to the water’s nutritional deficiency or presence of toxins in water runoff from residential and commercial applications of insecticides during the summer season. We added Tetramin Tropical Flakes, the standard larval mosquito diet in insectaries, to the microcosms. Food supplementation decreased pupation rate of *Ae. aegypti* and *Ae. albopictus* in the control (flowerpot water, Table 1), as a consequence of habitat over-nutrition by food addition. In contrast, food supplementation led to a marginally significant increase in the larval pupation rate of *Ae. aegypti* from 0% without food to 12.0% after adding food (*Z* = 1.82, *P* = 0.069, Fig. 6C and Table 1), but did not change the pupation rate of *Ae. albopictus*—the pupation rate remained at 0% (Fig. 6D and Table 1). These results suggest that the inability *of Ae. albopictus* larvae to develop into pupae in the summer USDS water was not entirely due to nutritional deficiency, but could have been caused by pesticide residues to which *Ae. albopictus* larvae were particularly sensitive.

To test this possibility, we conducted the third microcosm experiment by using USDS water in winter when there was no larval habitat treatment. We found food supplementation increased the pupation rate of *Ae. aegypti* from 2 to 61% (*Z* = 2.22, *P* < 0.05; Table 2; Fig. S2). For *Ae. albopictus,* the addition of food increased the pupation rate from 2 to 87% (*Z* = 1.96, *P* < 0.05; Fig. S2). The significant contrast observed in *Ae. albopictus* pupation rates between summer and winter in the USDS water with food supplementation (0% vs. 87%; *Z* = 2.02, *P* < 0.05) suggests that the presence of toxins in the USDS water in summer was also one of the driving forces for complete inhibition *of Ae. albopictus* larval development.

**Table 2:** Pupation rate and larval-to-pupal development duration of *Aedes aegypti, Ae. albopictus* and *Culex quinquefasciatus* larvae in winter underground water in 2019. Numbers in the bracket indicate 95% confidence interval.

Mosquito Species	Treatment	Pupation rate (%)	*P*	Pupation time (days)	*P*
*Ae. aegypti*	No food supplementation	2.0 (0, 5.9)	< 0.001	8.0 (5.9, 10.1)	> 0.05
	Food supplementation	61.0 (36.1, 85.9)		8.5 (8.1, 8.9)	
*Ae. albopictus*	No food supplementation	1.3 (0, 7.0)	< 0.001	8.7 (7.8, 9.7)	> 0.05
	Food supplementation	87.0 (76.7, 97.3)		8.3 (7.9, 8.8)	
*Cx. quinquefasciatus*	No food supplementation	1.9 (0, 5.8)	< 0.0001	13.5 (11.8, 15.2)	< 0.0001
	Food supplementation	82.0 (72.7, 91.3)		7.4 (7.2, 7.7)	

Although summer USDS water completely inhibited the development of invasive *Aedes* larvae, *Cx. quinquefasciatus* larvae demonstrated a 15.0% pupation rate, significantly lower than the 81% pupation rate observed in the flowerpot water (*Z* = 2.19, *P* < 0.05; Table 1). Food supplementation significantly increased its pupation rate from 15.0% to 63.0% in the summer USDS water (*Z* = 1.96, *P* < 0.05; Table 1), suggesting that USDS summer water was not highly toxic to *Cx. quinquefasciatus* larvae. Rather, summer USDS water conferred nutrimental constraint to *Cx. quinquefasciatus*. In winter, nutrimental constraint of the USDS water to *Cx. quinquefasciatus* was more severe due to its lower pupation rate in the USDS water without the addition of food and larger difference in pupation rate induced by food supplementation (82.0% vs. 2.0%; *Z* = 2.23, *P* < 0.05; Table 2).

## Discussion

This study was motivated by the question of the contribution of man-made USDS aquatic habitats to the productivity of invasive *Ae. aegypti* and *Ae. albopictus* pupae or adults in the urban environment of southern California. The USDS serves to drain run‐off water from homes, businesses, and streets in the urban environment, and also creates hard-to-treat stagnant water bodies in urban areas from thousands of kilometers of street gutters and underground pipes, numerous catch basins, and manhole access chambers^10,25^. Because stagnant water in the USDS in warm southern California has previously been shown to provide large numbers of suitable larval habitats predominated by *Cx. quinquefasciatus*^10^, we were interested in whether USDS offers productive larval habitats for newly invasive *Ae. aegypti* and *Ae. albopictus* to the region.

This study showed the rapid spread of invasive *Ae. aegypti* mosquitoes in Orange County, California, as evidenced by increasingly wider ranges of larval distribution and habitat positivity rates over a four-year period from 2016 to 2019. The largest expansion period occurred from 2017 to 2018. Although mosquito surveillance efforts were substantially enhanced since 2017, the observed *Ae. aegypti* spatial range expansion cannot be attributed to yearly differential sampling efforts: sampling efforts were comparable among 2017, 2018 and 2019. The distribution of *Ae. albopictus* was sporadic and limited in its abundance and spatial range: among the 6,072 aquatic habitats examined, *Ae. albopictus* larvae occurred in only 0.4% of aquatic habitats, whereas *Ae. aegypti* was found in 18.3% of the habitats. Invasive *Aedes* larvae were found mostly in containers, but 4.7% of the USDS habitats were positive for *Ae. aegypti* and 0% for *Ae. albopictus*. The USDS water did not attract or repel *Ae. aegypti* from laying eggs, but it attracted egg laying by *Ae. albopictus*, and was conducive for their egg hatching. Interestingly, USDS water inhibited larval development of *Ae. aegypti* and *Ae. albopictus*, particularly during the summer season when no larvae were able to develop into pupae.

Contrasting abundance and spatial distribution patterns between *Ae. aegypti* and *Ae. albopictus* in our study are remarkable given that both species were introduced/discovered in Orange County at the same time, 2015. The reason for this is not clear, but interspecific competition between *Ae. albopictus* and *Ae. aegypti* larvae is not likely a driving factor. Literature from other field studies in the eastern US, Asia, and South America suggest that *Ae. albopictus* larvae generally outcompete *Ae. aegypti* larvae, and *Ae. albopictus* has contributed to the declines in *Ae. aegypti* in southern North America^26^. A more plausible hypothesis is that the pattern of high *Ae. aegypti* abundance and occurrence in our study area may be determined by environmental conditions in the local area that help the survival of adult mosquitoes or eggs (e.g., desiccation) of *Ae. aegypti* but are less favorable to *Ae. albopictus*, rather than the result of competition among aquatic larvae. Further research is needed to combine population genetic analysis and field ecological studies to examine the founder effects and determine differential impacts of environmental conditions on *Ae. aegypti* and *Ae. albopictus*.

To circumvent the potential impact of mosquito colonization on their behavior and life history traits, the present study used *Ae. aegypti* and *Ae. albopictus* reared from field-collected eggs in the study area. To minimize the confounding effect of environmental factors such as temperature and predators, all studies were conducted in an insectary with climate regulation. Thus, although the experiments were not conducted in the field, the experimental setup enabled us to address the question of our interests. We found 4.1% of USDS habitats were positive for *Ae. aegypti*, and 0% for *Ae. albopictus* larvae, and USDS water was receptive to egg laying and permissive to egg hatching. However, USDS water was not permissive to invasive *Aedes* larval development and survival during the summer months when gravid females are available to lay eggs. Although winter USDS water allows larval development at a low population rate (~ 1.3–2%), the number of adult mosquitoes that can lay eggs is low at this time of the year. Therefore, the overall contribution of USDS aquatic habitats to the productivity of invasive *Aedes* mosquitoes is low. Other studies have previously reported the presence of invasive *Aedes* larvae in urban USDS habitats, e.g., *Ae. albopictus* larvae in urban stormwater catch basins and manhole chambers in China^27^, *Ae. aegypti* larvae in underground habitats in Brazil^28^, Puerto Rico^29,30^, Mexico^31,32^ and the United States^33,34^. One key unanswered question is what proportion of larvae can develop into pupae and emerge into adults. Mosquito surveillance programs that examine both larval and pupal abundance in the USDS water should address this question. Further, it is critical to determine whether the low success or complete failure of *Aedes* larval development into pupae in the USDS water as observed in the present study is unique to southern California or is a general phenomenon.

The mechanisms for the impact of USDS water on invasive *Aedes* larval development and survival are not clear. The zero pupation rate observed in *Ae. aegypti* and *Ae. albopictus* with summer USDS water can be attributed to nutrient deficiency, as well as poor water quality from low dissolved oxygen and nutrients, and abnormal electrical conductivity and salinity levels^10^ and perhaps residual insecticidal activity from pyrethroids (e.g., bifenthrin, a common household insecticide) found in Orange County’s urban runoff^35^. We tried to discern these possibilities by adding larval food to the microcosms and repeating the larval life table studies using USDS water from winter when there were few habitat treatments. A 12% pupation rate in *Ae. aegypti* and zero pupation in *Ae. albopictus* in microcosms with USDS water and food supplementation suggest that the USDS water was toxic to both species, with *Ae. albopictus* larvae suffering higher mortality. Water chemistry analyses by other investigators have demonstrated significant runoff in storm drain system from urban insecticide use in several counties in southern California^36,37^, suggesting pesticide runoff in USDS may be a widespread event. Considering the evidence of low pupation rates (~ 2%) for these two invasive *Aedes* species in the microcosm studies with winter USDS water when there was no active habitat treatment and thus low concertation of toxins, and drastically increased pupation rate after food supplementation, we conclude that nutrimental deficiency, toxins, and poor water quality in USDS summer water act together, leading to complete inhibition of larval development of *Ae. aegypti* and *Ae. albopictus* in the summer.

There were several limitations with the present study. First, we did not perform water chemistry analysis and thus could not pinpoint the precise reasons for the inhibition of invasive *Aedes* larval development in the USDS water. In addition to the reported low dissolved oxygen and nutrient content, abnormal electrical conductivity and salinity levels^10^, insecticides from many urban applications such as structural pest control, landscape maintenance, residential home and garden use, and mosquito control drain into the USDS^12,35,38,39^ and may fluctuate temporally and spatially as a consequence of dilutions by natural rainfall and irrigation, depending upon landscapes. Second, the microcosm experiments on the suitability of the USDS habitat for invasive *Aedes* egg laying, hatching and larval development used water mixtures from seven collection sites. The possibility of point-source effects on the observed results cannot be ruled out, in that a single sample that contained pesticide residue could be mixed into other samples, thereby affecting the results of the entire batch. However, the potential adverse effects from mixing water samples did not prevent development of *Cx. quinquefasciatus*. High levels of *kdr*-mediated pyrethroid resistance in Orange County populations of *Cx. quinquefasciatus* may have contributed to successful emergence of this mosquito in the microcosm experiments^40^. Future research will need to conduct water chemistry analysis to address this question. Third, we did not examine density dependence of larval survivorship in the microcosm studies. Nonetheless, our experiments using field mosquitoes under well-controlled conditions support the notion that USDS habitats are not major productive habitats for *Ae. aegypti* and *Ae. albopictus* in Orange County, California. The low quality of USDS water and slight attractiveness to ovipositing invasive *Aedes* may allow USDS habitats to act as ecological traps for invasive *Aedes* in southern California^41^.

In sum, this study examined the spread and aquatic habitat usage of two invasive *Aedes* species in the arid urban environment of southern California over a period of four years immediately after their introduction, and tested the suitability of USDS water for their egg laying, hatching, and larval development. Our results show rapid spread of *Ae. aegypti* in small containers in residential and commercial areas. Water from USDS habitats was receptive to egg laying and hatching, but completely inhibited successful development of invasive *Aedes* larvae to pupae. The notion that USDS habitats are currently not productive habitats for these invasive *Aedes* mosquitoes in southern California prompts including pupal productivity in the larval mosquito surveillance program. Finally, this work demonstrated that assessment of the impact of urbanization on the risk of vector-borne diseases requires careful examination of its impact on the environment and the ecology of disease vectors.

## Supplementary Information

Supplementary Information.

## Data Availability

The authors confirm that the data supporting the findings of this study are available within the paper and its supplementary information files.
