# Supplementary material for: Impact of underground storm drain systems on larval ecology of Culex and Aedes species in urban environments of Southern California
Source: Sci Rep. 2021 Jun 16;11:12667. doi: 10.1038/s41598-021-92190-3 (PMC8209202; doi:10.1038/s41598-021-92190-3)
Supplement: Supplementary file 1 — Supplementary Information. [file 41598_2021_92190_MOESM1_ESM.pdf]

Article Type: Research Article

Impact of Underground Storm Drain Systems on Larval Ecology of *Culex* and *Aedes* Species in Urban Environments of Southern California

Xiaoming Wang[1], Guofa Zhou[1], Daibin Zhong[1], Yiji Li[1], Stacia Octaviani[1], Andrew T. Shin[1], Timothy Morgan[2], Kiet Nguyen[2], Jessica Bastear[1], Melissa Doyle[3], Robert F. Cummings[2*], and Guiyun Yan[1*]

[1] Program in Public Health, University of California, Irvine, Irvine, CA 92697-4050, USA

[2] Orange County Mosquito and Vector Control District, Garden Grove, CA 92843, USA

[3] San Gabriel Valley Mosquito and Vector Control District, West Covina, CA 91790, USA

Correspondence: Robert F. Cummings: rcummings1026@gmail.com; Guiyun Yan, guiyuny@uci.edu

Table S1. Relative proportions of larval mosquito habitat types positive for any species identified from larval surveillance in Orange County, California, 2016-2019.

Habitat types	Year				
	2016	2017	2018	2019	Total
Container	275 (52.9%)	359 (17.1%)	728 (42.4%)	545 (31.4%)	1907 (31.4%)
Underground System	56 (10.8%)	403 (19.2%)	242 (14.1%)	353 (20.3%)	1054 (17.4 %)
Water Feature	124 (23.8%)	626 (29.8%)	413 (24.0%)	345 (19.9%)	1508 (24.8%)
Marsh	15 (2.9%)	307 (14.6%)	193 (11.2%)	274 (15.8%)	789 (13.0%)
Pool/Spa	39 (7.5%)	156 (7.4%)	55 (3.2%)	95 (5.5%)	345 (5.7%)
Creek	11 (2.1%)	248 (11.8%)	87 (5.1%)	123 (7.1%)	469 (7.7%)
Total	520	2099	1718	1735	6072

Note: Containers include flowerpots (vase), saucers, tires, bowls, boxes, buckets, dishes, tree holes, etc. Underground systems refer to breeding sources such as catch basins, drain/ditches, and manhole basins. Water feature includes ponds, fountains, bird baths, flood channels, gutters, and water reservoirs, etc. Marsh consists of fresh and salt water marshes.

Table S2. Summary of mosquito species and occurrence identified from larval mosquito surveillance in Orange County, California, 2016-2019

Genus	Species	No. Habitats	Proportion
Aedes	*Ae. aegypti*	1111	18.3%
	Ae. albopictus	25	0.4%
	Ae. notoscriptus	1	0.0%
	Ae. sierrensis	4	0.1%
	Ae. squamiger	21	0.3%
	Ae. taeniorhynchus	133	2.2%
	Ae. washinoi	17	0.3%
Anopheles	*An. franciscanus*	17	0.3%
	An. hermsi	182	3.0%
Culiseta	*Cs. incidens*	780	12.8%
	Cs. inornata	28	0.5%
	Cs. particeps	12	0.2%
	Cs. particeps	13	0.2%
Culex	*Cx. erythrothorax*	151	2.5%
	Cx. inornata	23	0.4%
	Cx. quinquefasciatus	3977	65.5%
	Cx. restuans	11	0.2%
	Cx. stigmatosoma	361	5.9%
	Cx. tarsais	1043	17.2%
	Cx. thriambus	23	0.4%
	Total	6702	100%

Table S3 Number of *Aedes aegypti* (*A.a.*) and *Culex quinquefasciatus (C.q.)* positive habitats in the three major larval habitat groups within the underground storm drain system from 2016 to 2019 in Orange County, California. Positivity rate was calculated as the proportion of larval habitats positive for *Ae. aegypti* or *Cx. quinquefasciatus* larvae among all habitats examined.

Habitat types	2016			2017			2018			2019			Total number and positivity rate over four-year period		
	n	*A.a.*	*C.q.*	n	*A.a.*	*C.q.*	n	*A.a.*	*C.q.*	n	*A.a.*	*C.q.*	*n*	*A.a. (%)*	*C.q. (%)*
Underground water retention vault	2	0	1	21	0	12	8	1	6	7	0	1	38	1 (2.6%)	20 (52.6%)
Underground catch basin/manhole	14	0	13	131	0	128	52	0	51	54	0	51	251	0 (0%)	243 (96.8%)
Underground pipeline/tunnel	40	0	38	251	8	202	182	10	153	292	26	219	765	44 (5.8%)	612 (80.0%)
Total	56	0	52	403	8	342	242	11	210	353	26	271	1054	45 (4.3%)	875 (83.0%)

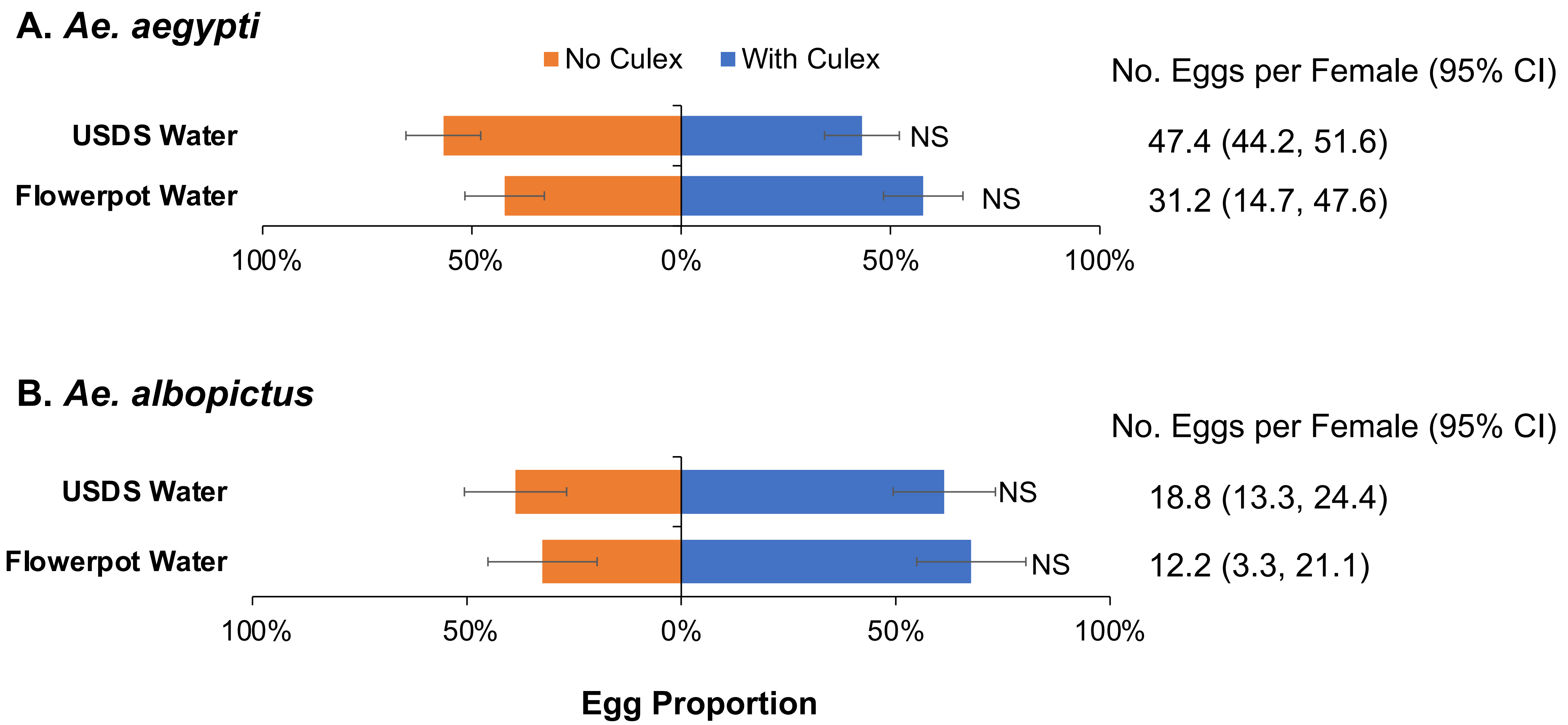

Figure S1. Effect of *Culex quinquefasciatus* larvae on oviposition preference for *Aedes aegypti* (A) and *Ae. albopictus* (B) in a two-choice oviposition preference test. Waters from underground storm drain systems and flowerpots collected in summer 2019 were tested.

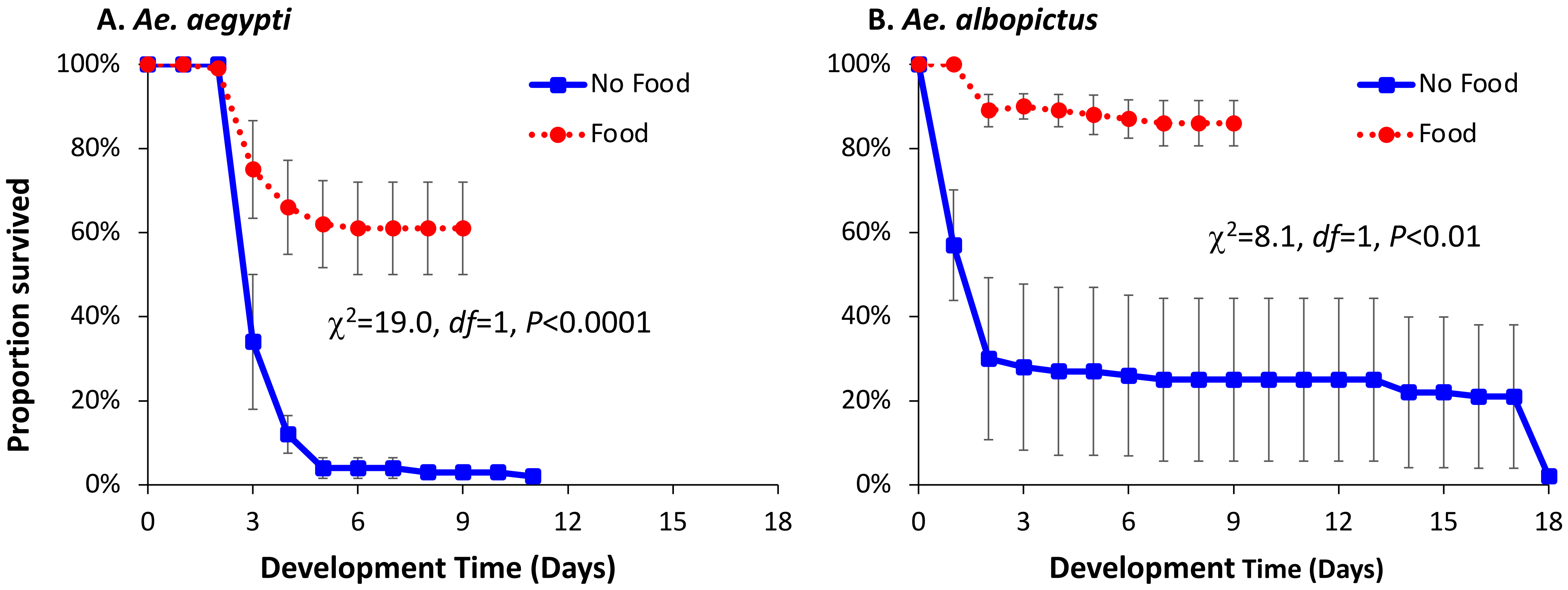

Figure S2. Effect of food supplementation on the survival of *Aedes aegypti* and *Ae. albopictus* larvae in microcosms with waters collected in winter, 2019, from underground storm drain systems and flowerpots in Orange County, California. A) *Aedes aegypti*; and B) *Ae. albopictus*. Chi-square and *P*-value of Kaplan-Meier survival analysis log-rank test is shown.
